# Psychotherapies for neurocognitive disorder due to alzheimer’s disease- the current level of evidence

**DOI:** 10.1192/j.eurpsy.2021.1122

**Published:** 2021-08-13

**Authors:** D. Vasile, O. Vasiliu, D. Vasiliu

**Affiliations:** Psychiatry, University Emergency Central Military Hospital Dr. Carol Davila, Bucharest, Romania

**Keywords:** Alzheimer’s disease, neurocognitive disorder, psychotherapy

## Abstract

**Introduction:**

Psychosocial interventions for Alzheimer’s dementia (AD) may increase patients quality of life and overall functioning, while it decreases caregivers burden. Therefore psychotherapies represent an important component of the case management, beside pharmacological approaches.

**Objectives:**

To review the current psychotherapeutic options available for patients diagnosed with AD, that may be added to their ongoing pharmacological treatment.

**Methods:**

A literature review was conducted through main electronic databases, and papers published between January 2000 and August 2020 were included in the analysis.

**Results:**

Cognitive stimulation therapy is based on general cognitive abilities training, with an accent over the social interaction, and it has been associated with significant improvement when compared to wait list or standard care. Reminiscence therapy, usually administered in a group format, focuses on past experinces, triggered by photos, newspaper fragments, music tunes etc., and according to a meta-analysis it may increase communicational, cognitive, and affective abilities. Validation therapy is based on a very empathic communication and tries to acknowledge patients perspective over the world, but the data to support its efficacy is limited for AD. Multisensorial stimulation is based on the assumption that stimuli deprivation is involved in the onset of anxiety, restlessness, insomnia etc, and may lead to short-term non-cognitive symptoms. Music therapy, art therapy, and animal-assisted therapy are also indicated, but more trials are needed to confirm their efficacy.

**Conclusions:**

A large number of psychotherapeutic interventions are explored for AD patients, but most of them have low levels of evidence.

